# Pregestational diabetes alters cardiac structure and function of neonatal rats through developmental plasticity

**DOI:** 10.3389/fcvm.2022.919293

**Published:** 2022-09-13

**Authors:** Md Jahangir Alam, Shravan Kumar Uppulapu, Vikas Tiwari, Bincy Varghese, Soheb Anwar Mohammed, Ramu Adela, Sudheer Kumar Arava, Sanjay K. Banerjee

**Affiliations:** ^1^Department of Biotechnology, National Institute of Pharmaceutical Education and Research, Guwahati, India; ^2^Non-communicable Diseases Group, Translational Health Science and Technology Institute (THSTI), Faridabad, India; ^3^Department of Pharmacy Practice, National Institute of Pharmaceutical Education and Research, Guwahati, India; ^4^Department of Pathology, All India Institute of Medical Sciences, New Delhi, India

**Keywords:** RNA sequencing, neonates, cardiac dysfunction, pregestational diabetes, heart development, developmental plasticity

## Abstract

Pregestational diabetes (PGDM) leads to developmental impairment, especially cardiac dysfunction, in their offspring. The hyperglycemic microenvironment inside the uterus alters the cardiac plasticity characterized by electrical and structural remodeling of the heart. The altered expression of several transcription factors due to hyperglycemia during fetal development might be responsible for molecular defects and phenotypic changes in the heart. The molecular mechanism of the developmental defects in the heart due to PGDM remains unclear. To understand the molecular defects in the 2-days old neonatal rats, streptozotocin-induced diabetic female rats were bred with healthy male rats. We collected 2-day-old hearts from the neonates and identified the molecular basis for phenotypic changes. Neonates from diabetic mothers showed altered electrocardiography and echocardiography parameters. Transcriptomic profiling of the RNA-seq data revealed that several altered genes were associated with heart development, myocardial fibrosis, cardiac conduction, and cell proliferation. Histopathology data showed the presence of focal cardiac fibrosis and increased cell proliferation in neonates from diabetic mothers. Thus, our results provide a comprehensive map of the cellular events and molecular pathways perturbed in the neonatal heart during PGDM. All of the molecular and structural changes lead to developmental plasticity in neonatal rat hearts and develop cardiac anomalies in their early life.

## Introduction

The prevalence of diabetes mellitus has been reported to be one of the common complications in pregnancy and has been estimated to affect 3.1–6.8% of pregnant women worldwide (1). Although a majority of diabetes-related pregnancy complications result from gestational diabetes (GDM), PGDM poses a more adverse impact on pregnancy outcome and maternal mortality than in women with GDM (2, 3). PGDM increases the risk of a variety of adverse pregnancy outcomes in women, such as congenital malformations, miscarriages, iatrogenic preterm delivery, preeclampsia, macrosomia, and stillbirth (4–6). Maternal hyperglycemia in PGDM is present during the initial phase of fetal development and organogenesis. Most of the PGDM-induced malformations occur during embryogenesis. In PGDM, both the unfavorable maternal environment and the genetic makeup ensue a complex process that induces damage to the embryo, the placenta, the fetus and the offspring (7, 8). Evidence suggests that offspring from diabetic mothers are at high risk for obesity, glucose intolerance, and cardiovascular dysfunction later in life (9–11).

Previous studies indicated that the degree of hyperglycemia during organogenesis is associated with an increased prevalence of diabetic embryopathy, including congenital cardiac and non-cardiac defects (12, 13). Among the various PGDM-induced congenital abnormalities, congenital heart defects (CHDs) are the most frequent (14, 15). During GDM or PGDM, cardiovascular malformations are among the most commonly observed malformations, which are about ten times more frequent than the average population (about 8.5% of cases vs. 0.8%) (16–18). The presence of various congenital malformations such as ventricular septal defects, the Tetralogy of Fallot and cardiomyopathy aortic stenosis is reported in neonates born from diabetic mothers (7, 17, 19, 20). According to a meta-analysis, CHDs were recorded in 3.6% of newborns of mothers with PGDM compared to 0.74% of babies born from non-diabetic mothers (7, 18). Among the diverge cardiovascular malformations, fetal cardiac hypertrophy is one of the pervasive consequences of hyperglycemia in the offspring (21).

The neonatal heart possesses developmental plasticity, which allows the heart to function normally. Terminally differentiated adult mammalian cardiomyocytes are hardly able to restore their function by cardiomyocyte renewal, but neonates can repair damaged tissue by proliferation. Moreover, in response to several stimuli such as maternal hyperglycemia, cardiomyocyte plasticity plays an essential role in cardiac adaptation to the adverse environment by remodeling the structure, function and metabolism of the heart [Gong et al. (22)]. As a consequence, alteration of key metabolic processes and developmental organization in the fetus results in developmental defects and altered growth trajectories. Experimental studies suggest that the hyperglycemic microenvironment during early embryogenesis may induce developmental heart defects by altering gene expression and cellular processes of the developing heart. However, the relationship between altered gene expression and cardiac phenotype during PGDM is unclear (23–27). It is of great importance to identify the metabolic and muscle contractile pathways that are defective in the early neonatal heart. These early defects in neonatal hearts may increase the risk of developing the cardiac disease in adulthood. Therefore, in the present study, we tried to understand the molecular defects in the neonatal heart by RNA-seq analysis and phenotypic correlation with these molecular changes.

## Materials and methods

### Animals study

All animal experimental protocols were approved by the Institutional Animal Ethical Committee (IAEC) of Translational Health Science and Technology Institute (THSTI), Faridabad-India or National Institute of Pharmaceutical Education and Research (NIPER), Guwahati-India and were carried out per regulations of IAEC and THSTI or NIPER guidelines on the care and welfare of laboratory animals. All animals were treated humanely and concerning the alleviation of suffering. Sprague–Dawley rats weighing 200–250 g were purchased from the National Institute of Immunology (NII), New Delhi, India. Animals were maintained at 22 ± 2^°^C temperature, 50 ± 15% relative humidity and 12 h of dark and light cycle, and had free access to water and diet. Animals described as fasted were deprived of diet for 12 h.

### Induction of pregestational diabetes in rats

Pregestational diabetes was chemically induced in female Sprague-Dawley rats (weight, 230-280 g) with Streptozotocin (STZ; Sigma-Aldrich, St. Louis, MO, United States). After fasting, the female rats were administered with a single intraperitoneal injection of a freshly prepared solution of streptozotocin in ice-cold citrate buffer (0.01 M; pH 4.5) at a dose of 40 mg/kg body weight. Animals were then monitored for the next 7 days for their blood glucose levels. A corresponding number of weight-matched animals were maintained as controls. The diabetic status of the animals was confirmed by measuring the blood glucose levels of the rats using a glucometer (Dr. Morepen^®^ Morepen Laboratories Ltd., India), with fasting blood glucose levels > 180 mg/dL considered to indicate diabetic conditions. After confirming diabetes, female diabetic rats were bred with male non-diabetic rats in trios (two females and one male). All females were mated with males overnight and checked for sperm plugs. The first day when the plug was visible was considered gestational day 1. After delivery of pups, 2 days old neonatal hearts were collected for the study. The collected hearts were washed in ice-cold PBS, blotted dry and put in liquid nitrogen, then stored at –80°C or in formalin for downstream analysis.

### The performance and measurements of electrocardiography

Two days after birth, recordings were conducted on pups from diabetic and non-diabetic rats after being anesthetized with isoflurane. The core body temperature of the animal was maintained at 37°C by a controlled heating pad (Homeothermic blanket control unit, Harvard Apparatus^^®^^). For the electrocardiogram (ECG), signal capture was accomplished with platinum electrodes inserted subcutaneously at three sites, namely positive (near the heart), negative (away from the heart) and ground (near right hind limb), and connected to an ECG amplifier and recorded for about 10 min (Powerlab, ADInstruments, Bella Vista, NSW, Australia). Motion-altered ECG signals and artifacts were removed before analysis. Recordings were analyzed for intervals with LabChart 8 software (ADInstruments, Bella Vista, NSW, Australia), and changes in the electrocardiographic parameters were calculated with the formula for rat QTc = QT/(RR/150)^1/2^ (28).

### Echocardiography of neonatal rat hearts

To investigate the cardiac structure and function, Fujifilm VisualSonics Vevo LAZR-X 3100 system (Fujifilm VisualSonics, Inc., Toronto, ON, Canada) was used. After anesthetizing neonatal rats, gently restrained on a three-axis micro-positioning stage to focus on the ultrasound precisely. After that, rats were placed supine on a heated pad to maintain a stable temperature (37°C). Echocardiographic examination was done with a high conductive ultrasound gel and using a linear transducer in 13-MHz (MX400) to obtain high-resolution two-dimensional and M-mode measurements. The pre-set mode used was “Mouse cardiology (small animal)” with the parasternal long-axis view (PSALX). The echocardiographic measurements were performed using a digital image analysis package (VevoLab version 3.2.2).

### Sample collection, RNA extraction and quality analysis

The rat pups were anesthetized with isoflurane on the second day of delivery. The pups were then sacrificed to isolate hearts. These heart samples were then snap-frozen in liquid nitrogen and stored at –80°C for later use. Total RNA from the hearts was extracted using TRIzol reagent (Invitrogen; Thermo Fisher Scientific, Waltham, MA, United States) and quantified at an absorbance of 260 nm using a spectrophotometer (NanoDrop 8000; Thermo Fisher Scientific, Inc., Waltham, MA, United States). Its integrity and quality were analyzed using an Agilent 2100 Bioanalyzer (G2939A A; Agilent Technologies, Santa Clara, CA, United States).

#### Library preparation

RNA sequencing libraries were prepared with Illumina-compatible NEBNext^^®^^ Ultra™ Directional RNA Library Prep Kit (New England BioLabs, Ipswich, MA, United States) at Genotypic Technology Pvt. Ltd., Bangalore, India. One microgram of total RNA was taken for mRNA isolation, fragmentation and priming. Fragmented and primed mRNA was further subjected to first- and second-strand synthesis. Purification was done using HighPrep magnetic beads (Magbio Genomics Inc, Gaithersburg, MD, United States) and then end-repaired, adenylated and ligated to Illumina multiplex barcode adapters as per the manufacturer’s protocol. Adapter-ligated cDNA was purified using HighPrep beads and was subjected to indexing to enrich the adapter-ligated fragments. The sequencing library (final PCR product) was purified with HighPrep beads and quantified by a Qubit fluorometer (Thermo Fisher Scientific, Waltham, MA, United States). Agilent 2200 Tapestation was used to determine library size and distribution.

#### RNA sequencing and differential gene expression analysis

The library products were prepared for sequencing with an Illumina HiSeq (Illumina, San Diego, CA, United States) at Genotypic Technology Pvt. Ltd., Bangalore, India. Four samples were obtained from the pups of the control and diabetic groups each to perform global gene expression analysis. In total, eight RNA-Seq libraries were constructed, and an average of 27 million clean reads were generated in each library. The RNA sequencing data have been deposited in GEO under accession code GSE196242. Data filtering was performed to obtain high-quality reads with 150 bp × 2, and reads were pre-processed to remove the adapter sequences and the low-quality bases (<q30). Pre-processing of the data is done with Cutadapt (29). An overview of average base quality is shown in Supplementary Figure 1A. Subsequently, ∼95.89% of the total reads were aligned and mapped to the rat genome as a reference^1^ (Rnor_6.0) (Supplementary Figure 1B). The pipeline used for RNA-seq analysis is given in Supplementary Figure 1C. In Supplementary Table 1, detailed statistics of mapped and unmapped transcripts are listed. Normalized expression levels for the identified genes were determined using the variable read per kilobase of exon per million mapped reads (RPKM) method (30). Fold-change (FC > 2) between the control and diabetic groups of rat pups were calculated for differential gene expression from the quadruplicate samples of both groups and the *p*-value < 0.05 (after FDR correction) is considered statistically significant. The volcano plot is used to show the relationship between significance [–log10(*p*-value)] on the *y*-axis and fold change of expression on the *x*-axis (Supplementary Figure 1D). The total number of differentially expressed genes (DEGs) was 1054, of which 68 were significantly upregulated and 271 were significantly downregulated.

#### Gene ontology and pathway enrichment analysis of differentially expressed genes

Metascape^2^ online bioinformatics tool was utilized for the gene ontology (Biological Processes, Molecular Functions and KEGG pathway analyses) and functional enrichment analysis of *Rattus norvegicus* genes (31). A minimum of three overlapped genes with an enrichment factor of 1.5 and *p*-value of 0.01 were considered for the hierarchical clustering (kappa score > 0.3) and network generation. Networks were visualized with Cytoscape (v3.7.2) (32). For identifying enrichment of specific terms of interest, an FDR (Benjamini and Hochberg method) of 5% was considered significant. Circos plot was used to depict the functional relationship between genes of the groups or between genes and ontology terms (33). The exported gene ontology results (top 25) from the DAVID V6.8^3^ (34) database were further plotted as dot plot using ggplot2 (V3.2.1) with the –log10(*p*-value) depicted by the color intensity and fold enrichment scale by circle size. After that, the R package GOplot [V1.0.2; (35)] was used to integrate the quantitative information with the significant and highly enriched terms (calculated by *z*-score).

### Gene regulatory network analysis and expression profile in the human heart

The transcriptional regulatory network analysis of DEGs was performed using the TRRUST database^4^. It is a prediction tool for human and mouse transcriptional regulatory networks (36) that uses a sentence-based text mining approach. The TRRUST database (version 2) contains 8444 transcription factor (TF)-target regulatory relationships of 800 human TFs. The expression profile of the predicted key regulators was analyzed using tissue-specific RNA-seq data of the Genotype-Tissue expression (GTex, v7 release) project (37).

### Histopathology

Two-day-old neonatal hearts were subjected to histopathology to observe fibrosis and cell proliferation. Whole heart tissues were excised and cleaned with ice-cold PBS, then fixed in 4% formalin, routinely processed and embedded in paraffin. Paraffin sections were cut into 5 μm thick sections and mounted on glass slides, then stained with hematoxylin and eosin (H&E), Masson trichrome stain and examined under a light microscope (Nikon Eclipse Ti). For the measurement of cardiomyocyte size, ImageJ software was used to measure the cardiomyocyte diameter at the level of the nucleus from H&E stained micrographs.

### Gene expression measurement by the polymerase chain reaction

RNA was isolated from neonatal heart tissues of both groups using the TRIZOL reagent (Sigma-Aldrich, St. Louis, MO, United States) following the manufacturer’s protocol. Quantification of RNA was done with the NanoDrop Spectrophotometer (Thermo Scientific, Waltham, MA, United States). DNase treatment was done before cDNA synthesis using 1 μg RNA with the SuperScript III Reverse Transcriptase (Takara Bio, San Jose, CA, United States). A reverse transcriptase-polymerase chain reaction (PCR) was carried out using the EmeraldAmp PCR Master Mix and using the following primers for target genes: Forward primer, 5′-GGAAGATCATGGAGCAGTCG-3′ and Reverse primer, 5′-GTCGGGATAATCAGCCATGT-3′ for Sox11 and Forward primer, 5′-CCACCGAGCTATCCACTCAT-3′ and Reverse primer, 5′-GTCCGGTTTCAGCATGTTTT-3′ for Mmp9. RNA expression levels were normalized using ribosomal protein L32 (Rpl32) as a reference gene using the following primer pair: Forward primer, 5′-AGATTCAAGGGCCAGATCCT-3′ and Reverse primer, 5′-CGATGGCTTTTCGGTTCTTA-3′.

### Immunohistochemistry for the measurement of cell proliferation

Neonatal rat hearts were harvested and fixed in formalin and paraffin-embedded for immunohistochemistry analysis. After antigen retrieval with 10 mM sodium citrate buffer (pH 6.0) at 80°C for 10 min, endogenous peroxidases were blocked by 3% hydrogen peroxide in PBS for 10 min. The slides were then incubated overnight with primary antibodies against Ki-67 (markers of proliferation) at 4°C in a humidified chamber and incubated with horseradish peroxidase (HRP)-conjugated secondary antibodies at 1:100 dilutions for 30 min at 37°C and visualized by 3,3′-diaminobenzidine tetrahydrochloride reagent. The sections were counterstained with hematoxylin and digitally imaged. The antibodies used were as follows: anti-Ki-67 (Bio SB, Cat. No. BSB-3767) at the dilution of 1:100 and HRP-linked anti-rabbit IgG (Cell Signaling, 7074S).

### Protein expression by western blotting

Protein samples were prepared by homogenizing 50 mg of tissue from neonatal hearts of both control and PGDM groups with 500 μL of RIPA buffer with added protease (Cat. No. 11836170001) and phosphatase inhibitor (Cat. no. 4906837001). Tissue lysates were centrifuged at 12,000 rpm at 4°C, and the supernatant was collected then protein levels were measured by Bradford protein assay using BSA as a standard. Proteins were separated by 10% (v/v) SDS-PAGE with G-SNAP (in-gel protein visualization reagent; GCC Biotech; Cat. No. GPSN 101) by loading 30 μg/well and the protein bands were transferred onto the 0.45 μM pore size nitrocellulose membranes (Cat. No. 1620115; Bio-Rad). Non-specific binding of the primary antibody was prevented by blocking the membranes with 3% BSA in TBST buffer. The antibodies used were as follows: anti-Sox11 (Abclonal, Woburn, MA, United States; Cat. No. A17945) at the dilution 1:1000, anti-Pbx1 (Abclonal, Woburn, MA, United States; Cat. No. A0124) at the dilution 1:1000, anti-Foxo3a (Abclonal, Woburn, MA, United States; Cat. No. A0102) at the dilution 1:1000, anti-Pitx2 (Abclonal, Woburn, MA, United States; Cat. No. A1870) and HRP-linked anti-rabbit IgG (Abclonal, Woburn, MA, United States; Cat. No. AS014) at the dilution 1:5000. Bands with bound antibodies were visualized using enhanced chemiluminescent using the Fusion Solo S imaging system (Vilber, France). The stain-free gel was used as the loading control and the ImageJ software system (NIH Image, National Institutes of Health, United States) was used to quantify the signal intensity of the protein bands.

### Statistical analysis

Data in the present study is reported as the mean ± standard error of the mean. Mean differences between the study groups were analyzed by *t*-test or one-way analysis of variance (ANOVA) among groups, followed by the Bonferroni multiple comparison test. A significance level is assumed if *p* < 0.05. Statistical analysis was performed in GraphPad Prism 8.2.1 (279) (Graph Pad Software Inc., San Diego, CA, United States).

## Results

### Electrocardiographic parameters were altered in neonates from streptozotocin-induced diabetic rats

**FIGURE 1:**
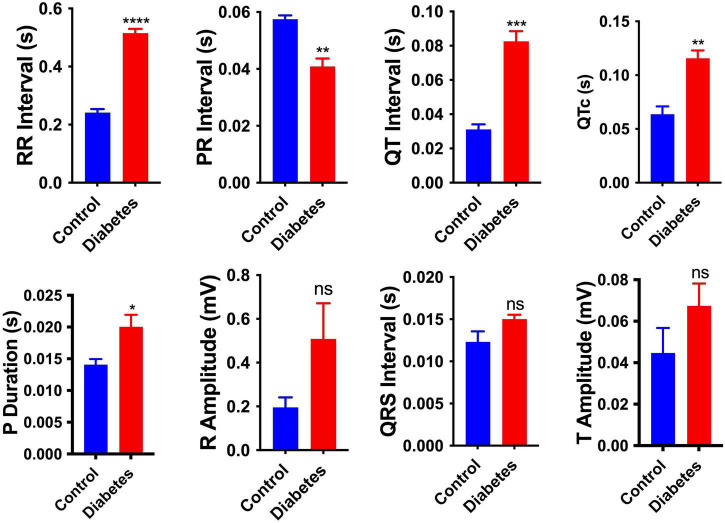
Electrophysiological changes in the neonatal hearts. Changes in the electrocardiography parameters of the 2-day neonatal hearts. QTc, corrected QT interval. Data are shown as Mean ± SEM (*n* = 5), *p < 0.05, **p < 0.01, ***p < 0.001, ****p < 0.0001. ns, no significant.

To investigate the effect of pregestational diabetes on the electrophysiology of neonatal rat hearts, electrocardiography (ECG) of the neonates was performed at 2 days of birth, and changes in the electrocardiographic parameters, i.e., RR interval, PR interval, QT interval, P duration, corrected QT intervals (QTc), R amplitude, T amplitude and QRS interval were recorded (Supplementary Table 2). As shown in Figure 1, the pups from diabetic mother had significantly longer RR interval (0.241 ± 0.012 vs. 0.515 ± 0.014 s), QT interval (0.031 ± 0.003 vs. 0.083 ± 0.006 s), P duration (0.014 ± 0.0009 vs. 0.02 ± 0.002 s), QTc (0.064 ± 0.007 vs. 0.116 ± 0.007) and shortened PR interval (0.058 ± 0.001 vs. 0.041 ± 0.003) compared to pups from healthy mother. However, no significant changes were observed in the parameters like QRS interval, R amplitude and T amplitude between the two groups of rats. Increased QT interval denotes delay in ventricular repolarization. However, the PR interval was shortened, which shows early atrial conduction. A significant increase in P wave duration represents longer atrial depolarization in neonates from diabetic rats in comparison to the control rats suggesting conduction delay between the left and right atrium.

### Cardiac systolic/diastolic dysfunction and structural remodeling are more prominent in neonates from streptozotocin-induced diabetic female rats

**FIGURE 2:**
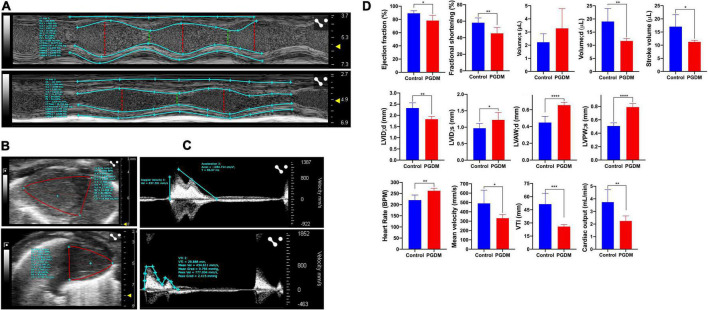
Echocardiography of the two-day neonatal hearts. Transthoracic echocardiographic analyses were performed using the Vevo LAZR-X ultrasound system: Representative images of M-mode **(A)**, B-mode **(B)** and pulsed-wave doppler **(C)** traces of the left ventricles are shown, and different functional hemodynamic parameters were evaluated **(D)**. Data are shown as Mean ± SD (*n* = 6), *p < 0.05, **p < 0.01, ***p < 0.001, ****p < 0.0001.

*In vivo*, systolic and diastolic functions were assessed from M-mode ultrasound echocardiography. Representative traces of M-mode, B-mode echocardiogram and pulsed-wave Doppler (PWD) are shown in Figures 2A–C, respectively. A modest diabetes-induced reduction (*p* < 0.01) in ejection fraction (11.1% decrease) and fractional shortening (12.9% decrease) was evident in the neonates from diabetic females only (Figure 2D). A trend for the diabetes-induced decreased end-diastolic volume of the neonatal hearts was evident (38.93% decrease, *p* = 0.01, Figure 2D). These data suggest that mild diastolic dysfunction is evident in neonates from STZ-induced diabetic rats. Anatomic changes in heart geometry were examined by measuring anterior and posterior wall thickness (LVAW and LVPW) from M-mode echocardiography. Left ventricular posterior wall thickness at end-diastole (LVPWd) was similar in both the groups but a significant (*p* < 0.0001) increase in Left ventricular posterior wall thickness at end-systole (LVPWs) was observed in diabetic groups. Left ventricular anterior wall thickness at end-systole (LVAWs) was similar in both the groups but a significant (*p* < 0.0001) increase in anterior wall thickness at end-diastole (LVAWd) was observed. The observed increase in the LVPWs and LVAWd is further confirmed by a significant increase in cardiomyocyte size in the Hematoxylin-eosin-stained sections (Supplementary Figure 2A). A modest but significant (21.37% increase, *p* < 0.05) increase in left ventricular internal diameter (LVIDs) was evident in the neonates from diabetic females at end-systole (Figure 2D). As a functional consequence, the neonates from diabetic mothers produced a significantly (*p* < 0.05) lower stroke volume and cardiac output as compared to normal rats.

Interestingly, the neonatal rats from diabetic mothers exhibited a faster heart rate than the neonates from normal mothers. Left ventricular outflow tract velocity time integral (LVOT VTI) is a measure of cardiac systolic function in terms of stroke volume (SV) and cardiac output (CO). As shown in Figure 2D, STZ-induced PGDM is statistically associated with a lower LVOT VTI (*p* < 0.001), SV and CO in their neonates, which further indicates the adverse outcomes in left ventricular failure. No significant diabetes-induced change in ventricle weight was detected in the neonates (Supplementary Table 3). Collectively these findings suggest that STZ-induced diabetes in female rats predisposes their offspring to cardiac dysfunction as characterized by wall thickening, chamber dilation and less contraction.

### Enrichment among genes with altered expression in the neonatal rat hearts

**FIGURE 3:**
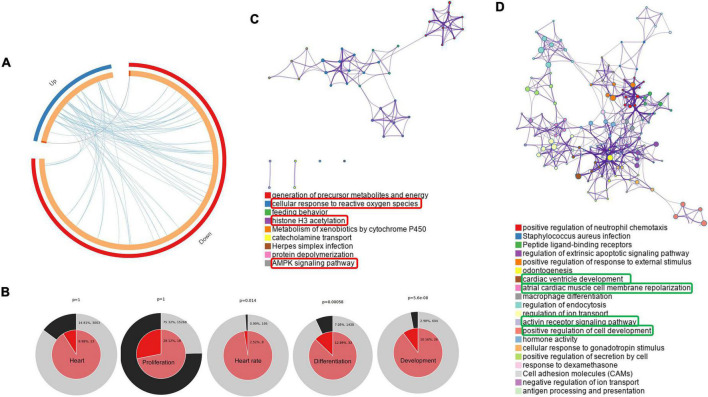
Networks of differentially regulated genes in the neonatal rats. **(A)** Gene overlap analysis by Circos plot shows the overlap between genes and functional categories based on two input gene lists: Up-regulated genes (blue arc) and the genes that were down-regulated in neonatal rats (red arc). **(B)** Enrichment of heart development processes in neonatal rats. Enrichment of genes matching membership terms: heart, heart rate, development, differentiation and proliferation in the GO Biological Processes, KEGG Pathway. The outer pie shows the number and percentage of genes in the background associated with the membership (in black); the inner pie shows the number and percentage of genes in the individual input gene list associated with the membership. Hierarchical cluster of statistically enriched and significant terms **(C,D)**. Network layout from a subset of representative terms from the whole cluster. Each pie sector is proportional to the number of hits originating from a gene list. Similar clusters of upregulated **(C)** and downregulated **(D)**. The term with the most significant enrichment was selected for each cluster to describe its ID (Lower panel).

In our study, we performed a comprehensive enrichment analysis using Metascape and Cytoscape based on DAVID gene ontology and KEGG pathway, then visualized using GOplot, which provides a novel understanding of the molecular mechanisms behind the progression of PGDM and its impact on neonatal hearts. Apart from DGE analysis, we also performed phenotypic studies of the neonatal hearts of diabetic female rats to explore the structural and functional changes using ECG, echocardiography and histopathology. In the attempt to classify the changes in gene ontology, which may give a glimpse of molecular changes in the neonatal hearts of diabetic rat mothers, we identified statistically significant changes in the biological process (BP), molecular function (MF) and KEGG pathway sets by bioinformatic analysis using Metascape tools (ANOVA *p* < 0.05; fold change threshold 1.5, Figure 3). The circos plot depicted in Figure 3A shows that there is substantial functional overlap among the input gene lists indicated by the blue links. As indicated by purple lines, there is minimal overlap among the input gene lists. Significant enrichments in heart rate, development, and differentiation were evident in DE genes (normal vs. diabetic neonates) under both experimental conditions (Figure 3B). Next, we selected a sub-group of representative GO terms from the whole cluster obtained and then, based on similarity scores (>0.3), the cluster was converted into a network layout. The enrichment network in Figures 3C,D is displayed as pies in the nodes, which is proportional to the number of hits originating from a gene list. One term from each cluster is selected to have its term description shown as the label. Several of the clusters in the network from upregulated genes labeled as “cellular response to reactive oxygen species,” “histone H3 acetylation,” and “AMPK signaling pathway” were compromised (Figure 3C). Several clusters in the network from downregulated genes were associated with pathways such as cardiac ventricle development, atrial cardiac muscle cell membrane repolarization, activin receptor signaling pathway, and positive regulation of cell development (Figure 3D).

### Gene ontology and network mapping reveal a connection between pregestational diabetes and cardiac developmental defects in neonatal rats

**FIGURE 4:**
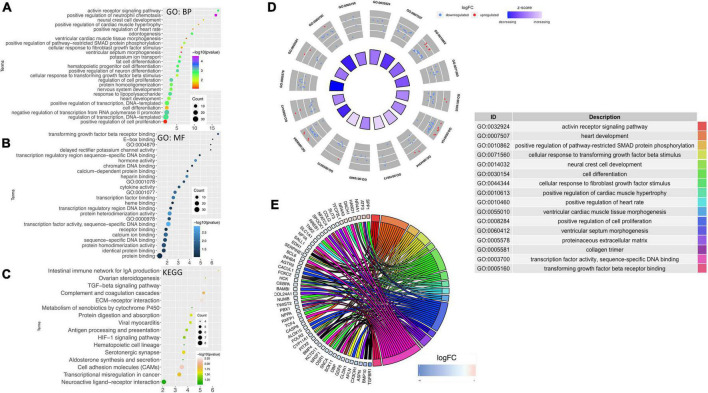
Gene ontology and network mapping. Bubble diagram of enriched terms of DEGs by DAVID database relating to change in the **(A)** biological process, **(B)** molecular function and **(C)** KEGG pathways. Significant pathways with *P*-values < 0.05 were plotted by the ggplot2 (R package). **(D)** GOCircle plot; the inner ring is a bar plot where the bar height indicates the significance of the term (–log10 *p-value*) and the color indicates the *z*-score. The outer ring displayed scatterplots of the expression levels (logFC) for the genes in each term. **(E)** GOChord plot of the relationship between the list of selected genes and their corresponding GO terms, together with the logFC of the genes. The left half of GOChord displayed whether the gene was up-or down-regulated. The right half represented different GO terms with different colors. A gene was linked to a certain GO term by the colored bands.

Using DAVID, the results of the GO analysis showed that major changes in DEGs were associated with biological processes and mainly enriched in the heart development pathway and cardiac structure and its function (Figure 4A). Concerning the molecular function, DEGs were significantly enriched in transforming growth factor ß (TGF-ß) receptor binding and transcription factor activity (Figure 4B). Analysis of KEGG pathways showed that the topmost biological pathways associated with DEGs were “TGF-ß signaling pathway,” “HIF-1 signaling pathway,” “cell adhesion molecules,” and “ECM-receptor interaction” (Figure 4C).

As the heart tissues were used for the RNA-seq study, our inquiry was limited to diseases of the cardiovascular system. Thus, investigations were conducted to determine STZ-induced PGDM in female rats, which presented a pattern of gene expression consistent with cardiac defects. To visualize the most interesting pathways related to the cardiovascular system, a GOcircle plot was generated using the GOplot of the R package (Figures 4D,E). The GOCircles shows the summary of the principal biological processes and molecular functions perturbed in the neonates of diabetic rat. The outer circle represents a scatter plot for each GO term with logFC calculated for the genes. The red dots and the blue dots represent upregulated and downregulated genes, respectively (Figure 4D). In the lower panel, Circos plot from selected genes and their respective links with certain GO terms depicted by different colored ribbons (Figure 4E).

Based on PubMed literature and Figure 4, significantly modulated genes (Supplementary Table 3) which have an association with biological processes related to heart development at cellular and organ level, cardiac conduction system, cardiac remodeling and vasculature development were further categorically summarized in Table 1. Most of the genes that were down-regulated have essential roles during embryonic development (Nr2f1, Bambi), heart developmental processes such as the formation of the second heart field (Pitx2 and Twist2) and development of the outflow tract (Foxc2, Pbx1, Pitx2, Sox11). A decreased level of Sox11 mRNA is observed by the PCR method and decreased levels of Pitx2, Pbx1, Foxo3a, and Sox11 protein are observed by western blotting, which further support an alteration in heart development (Supplementary Figures 2B,C). At the cellular level, genes associated with cardiomyocyte differentiation (e.g., Bmp10, Twist2) and cell migration (e.g., Foxc2, Cldn1) were downregulated. Cell proliferation-related genes were either down-regulated or up-regulated (i.e., Dach1 and Hand1). The down-regulated genes have either positive (e.g., Inhba) or negative effects (e.g., Agtr2) on cell proliferation. The structure and function of the heart in the neonates were also compromised, as evidenced by alteration of gene expression associated with the cardiac conduction system (Bmp4 and Pitx2), cardiac remodeling (Bambi, Bmp10, and Spp1) and vasculature development (Slit3, Bmp4, Pbx1, and Dach1).

**TABLE 1:** Categorical summarization of significantly modulated genes associated with heart structure and functions during development.

Biological processes (Heart structure and function)	Down-regulated genes		Up-regulated genes
	Positive impact	Negative impact	
First heart field formation	SALL1		HAND1
Second heart field formation	SALL1, PITX2, TWIST2		
Outflow tract development	FOXC2, PBX1, PITX2, SOX11, TGFBR1, BMP4		
Cardiomyocyte differentiation	PF4, GDF6, APLN, KCTD11, TWIST2	SALL1	HAND1
Cell proliferation	BMP10, INHBA, TGFBR1, BAMBI	AGTR2, KCTD11	DACH1, HAND1
Cell migration (Neural crest)	FOXC2, CLDN1		DACH1
Embryonic development	NR2F1	BAMBI	
Cardiac conduction system	BMP4, PITX2		
Cardiac remodeling	TWIST2	BMP10 (Anti-fibrosis), CASP8, PITX2, BAMBI	SPP1 (fibrosis)
Vasculature and angiogenesis	SLIT3, NFATC4, BMP4, PBX1	CD4	DACH1, NR4A1
Heart structure and function	AGTR2, TNNT3, EDN1, OSR1		

Furthermore, ontology analysis of the genes showed that the downregulated genes were involved in the activin receptor signaling (e.g., Inhba) and BMP signaling pathway that may impact cardiac development and remodeling. Additionally, reactive oxygen species, histone acetylation and regulation of AMPK cascade terms were upregulated. The key enzyme catalyzing the rate-limiting reaction of glycolysis and one of the targets of AMPK, Pfkfb3 (6-phosphofructo-2-kinase), was upregulated, suggesting higher energetic reliance of neonatal rats on glycolysis. In the neonatal heart of diabetic female rats, Hdac6 and Mff were also upregulated (Figure 4), which may contribute to the increased production of reactive oxygen species and ischemic injury in the myocardium. Altogether, ontology analyses suggest that STZ-induced pregestational diabetes in female rats results in the decreased expression of anti-fibrosis-related genes and cardiac development-related genes predominantly in their offspring (Table 1).

### Pregestational diabetes induces fibrosis and cell proliferation in neonatal rat hearts

**FIGURE 5:**
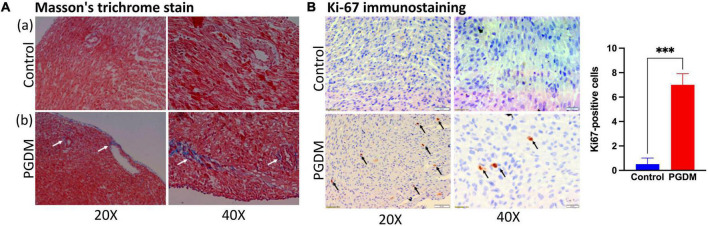
Histopathology of the neonatal hearts. **(A)** Photomicrograph of hearts after Masson’s Trichrome stain (*n* = 3): (a) Neonatal rat heart sections from non-diabetic females showing no fibrosis. (b) Neonatal rat heart sections from diabetic females showing fibrosis (collagen deposition; see arrows). **(B)** Ki-67 immunostaining of neonatal rat hearts from diabetic mothers showing increased cell proliferation (see arrows). Quantification of the ki-67 positive cells is shown in the right panel (*n* = 3).

To determine whether the prominent cardiac dysfunction in neonates from diabetic females corresponded to more marked structural remodeling, myocardial collagen content was assessed in the hearts of the neonates. We also found down-regulation of genes that negatively regulate the cardiac fibrosis pathway and alter the cell proliferation in the hearts of neonatal rats (Table 1). Moreover, the hyperglycemic condition during fetal development may directly activate a fibrogenic program by activating the transforming growth factor-β/Smad signaling in the fibroblasts, which may lead to the accumulation of collagen and matricellular macromolecules (38). Thus, slides of frozen heart tissues from pups of the non-diabetic and diabetic mothers were prepared, and the total collagen fiber fractions were determined by Masson’s trichrome staining. As shown in Figure 5A, the fraction of collagen fiber was higher in the STZ group compared to the control group, indicating the presence of cardiac fibrosis in the pups of the diabetic female rat. Since we observed a moderate degree of nucleomegaly along with frequent mitosis in the cardiomyocytes. Therefore neonatal heart tissues were also stained with anti-Ki-67 antibody to measure the proliferation index, which showed more frequent proliferation in the diabetic group of neonatal hearts than in the control groups (Supplementary Figure 2A and Figure 5B). The extent of fibrosis was further checked by measuring the Mmp9 mRNA level by PCR (Supplementary Figure 2B). We did not find a statistically significant change that might be due to the sporadic nature of fibrosis in neonatal hearts. The alteration in heart development in neonates from diabetic mothers was further checked by measuring the protein levels of crucial TFs Pitx2, Pbx1, Foxo3a, and Sox11 by western blotting (Supplementary Figure 2C). A significant decrease in the three TFs i.e., Pitx2, Pbx1, and Foxo3a confirms the finding by RNA-seq. Altogether, histopathological data and, mRNA and protein expression data corroborated with the RNA-seq data as we found altered heart development and function, increased cardiac remodeling (fibrosis) and cell proliferation in the neonatal hearts of diabetic female rats.

### Gene regulatory network analysis of the differentially expressed genes revealed human transcription factor involved in heart development and function

**FIGURE 6:**
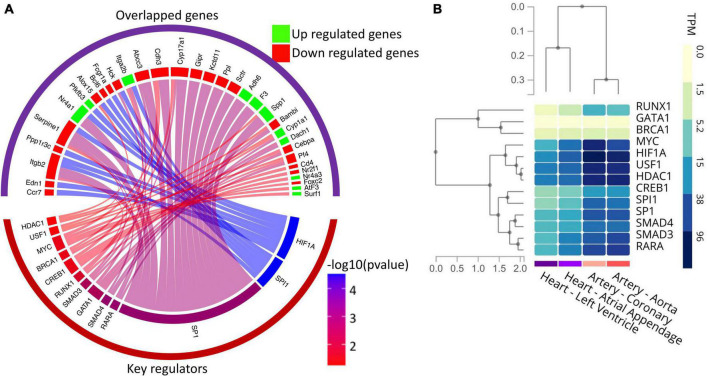
Gene regulatory network analysis of the DEGs and expression profile of key regulators in the human heart. **(A)** Circos plot showing the association among differentially expressed genes and their key regulators, including transcription factors. The upper group of sectors represents overlapped genes, while the lower one represents the key regulators. The width of individual sectors of corresponding key regulators reflects the number of links connecting to the DEGs, while their color indicates the significance of interaction. **(B)** Gene expression (in TPM) of human key regulators in the cardiovascular tissues as analyzed by RNA-seq data available in the GTex database.

Since our results indicate the significant alteration in the expression of transcription factors in diabetic hearts vs. normal hearts, we further explored potential key regulators, including transcription factors of the DEGs identified in the RNA-seq study using the TRRUST database. Thus, DEGs were submitted to the TRRUST database, and 43 genes were mapped as the key regulators of the included genes. Out of 43 key regulators, 13 TFs were significantly (hypergeometric *p* < 0.05) associated with 33 DEGs. Significantly associated regulatory pairs were visualized in a circos plot using the circlize package of R software (v. 0.4.13) (Figure 6A). As shown in Figure 6A, the transcription factors of human counterpart viz. HIF1A, SPI1, SP1, RARA, SMAD4, GATA1, SMAD3, RUNX1, CREB1, BRCA1, MYC, USF1, and HDAC1 have significant regulatory interaction with the genes whose expression levels were altered in the neonatal hearts from diabetic rats. To confirm the tissue specificity of the human TFs, we explored the expression of the TFs in cardiovascular tissues, namely the left ventricle, atrial appendage, aorta and coronary artery, using the GTEx database. The results showed that most TFs are expressed in the cardiovascular tissues. Taken together, the results suggest that human TFs may regulate the genes in the human heart and may be responsible for the phenotypic changes (Figure 6B).

## Discussion

Pregestational diabetes adversely impacts embryonic development, which results in the risk of both maternal and fetal malformations. Prolonged exposure to PGDM poses a high risk of developing cardiovascular disease in both mother and their offspring (39). In the current study, pregestational diabetes was developed chemically using streptozotocin (STZ) in female Sprague-Dawley rats. All the STZ-treated rats showed induction of diabetes characterized by hyperglycemia (Supplementary Figure 2D). The study suggests that diabetic patients have a high incidence of diabetic cardiomyopathy, as evidenced by complex changes in the electrical-physiological and conduction properties of the heart (40). In diabetic cardiomyopathy, cardiac remodeling is characterized by conduction system impairment and mechanical abnormalities, leading to arrhythmia (41, 42). Cardiovascular complications are the prime causes of mortality in patients with both type 1 and type 2 diabetes mellitus. In our study, neonatal rats from diabetic females showed altered ECG parameters as evidenced by reductions in the P duration, and prolongation of RR and QT interval. A significant decrease in PR interval was also observed (Figure 1). These ECG findings indicate disturbance in the conduction system of the heart, which may be caused by the change in the fetal microenvironment, such as glycation of proteins and hyperglycemia-induced ROS generation. Heightened and prolonged oxidative stress causes damage to the cell membrane and loss of its function, leading to cardiac conduction disturbances (43). The QT interval prolongation may also be related to cardiac dysfunction such as arrhythmias and sudden cardiac death (44).

The diabetes-associated myocardial dysfunction may arise due to metabolic alterations (45), macro-and microvascular disease (46), and myocardial fibrosis (38). Evidence suggests that early systolic LV dysfunction and dilatation of the left ventricle were detected in STZ-induced diabetes using M-Mode and bi-dimensional echocardiography (47–49). Thus, we sought to study the effect of pregestational diabetes in female rats on their neonatal pups using echocardiography and histopathological study to determine structural and functional changes in the hearts. The echocardiographic findings demonstrated that the hearts of the neonatal rats from diabetic females have systolic/diastolic dysfunction and structural remodeling as characterized by reduced ejection fraction and fractional shortening (Figure 2). Reduced myocardial contractility in diabetes may result from abnormal carbohydrate and lipid metabolism and their transport due to decreased insulin levels, the altered calcium handling by the sarcoplasmic reticulum in the heart (50) and an accumulation of toxic fatty acid intermediates inside the cardiomyocytes (45). These, in turn, can lead to progressive loss of myofibrils (51), causing myocyte hypertrophy, induction of fibrosis, and consequent detrimental effects on myocardial contractility.

After confirming the phenotype in the neonatal heart, we were interested to know more details about the molecular changes that may be responsible for the cardiac abnormalities in the early heart. Numerous studies have focused on the effects of maternal diabetes/gestational diabetes on the development of the whole embryo and embryonic heart (4, 5, 7, 8). Although studies suggest that pre-existing diabetes has more adverse effects during pregnancy and post-natal life, no mechanistic study to demonstrate the molecular mechanism leading to CHDs has been performed to date. We suspect that hyperglycemic condition creates an adverse microenvironment in the fetus that causes changes in gene expression, which in turn leads to alteration in the developmental pathway. In the present study, we investigated the global molecular signature associated with the mechanism of diabetes-induced structural and functional dysfunction using RNA-Seq analysis in neonatal rats. Our results showed that a total of 1054 genes were differentially expressed. Moreover, bioinformatics analysis demonstrated that 68 genes of the DEGs were significantly upregulated, and 271 genes were significantly downregulated in the hearts of neonates from diabetic female rats. A large number of the DEGs are transcription factors/activators which have predominant roles in heart development and differentiation and heart electrical conduction (Table 1). All the changes in gene expression are essential for the adaptation of the heart, which may also be defined as cardiac plasticity.

Manifestation of cardiac plasticity starts with embryonic heart development or cardiogenesis and occurs mainly by cell proliferation before they get terminally differentiated. The heart is the first and major organ developed in the fetus; thus, it is more susceptible to various teratogens, which may result in fetal mortality and the development of congenital heart diseases (CHDs). CHDs are described as abnormal development of the heart and/or the great vessels before birth, resulting in structural malformation such as ventricular septation defects and transposition of the great arteries, and functional abnormalities, such as alteration in electrical conduction. Numerous evidence suggests that these abnormalities are often associated with mutations in several transcription factors that control normal heart development (52, 53). For the formation of a four-chambered heart, activation of a battery of transcription factors, such as Tbx5, Nkx2-5, Gata4, Hand1 etc., is required, which modulates the remodeling events, i.e., chamber formation, septation, and valve development (54). Hyperglycemia and abnormal insulin signaling may lead to cardiac defects by impairing the expression of key regulatory genes as well as posttranslational modification of transcription factors that may result in the modulation of crucial target genes (55). A nationwide cohort study showed that pregestational diabetes represented a four-fold increase in CHD in their offspring (56). Our enrichment analysis (Figures 3, 4 and Table 1) suggested that regulatory processes related to embryogenesis and organ development, especially heart development, became apparently downregulated under the hyperglycemic condition present during the gestational phase. The upregulated transcription factors Dach1 and Hand1 observed in the present study have crucial roles in coronary artery development and heart development (proliferation and differentiation), respectively (57, 58). Other upregulated TFs like NR4A1 and NR4A3 are members of immediate early response genes, which can translocate from the nucleus to mitochondria, thereby inducing apoptosis of cells. NR4A3 is an indispensable gene for beta-cell function and insulin secretion (59). However, most of the transcription factors are downregulated, which include Foxc2, Nr2f1, Cebp, Sall1, pitx2, Osr1, Nfatc4, Sox11, Pbx1, TGFBR1, and Twist2. These TFs have important functions during cardiac neural crest cell migration and outflow tract morphogenesis, embryonic development, epicardial activation during heart development and injury, cardiac differentiation and first heart field and second heart field specification, morphogenesis of cardiac valve, and patterning of great arteries and vasculature (60–70). Therefore, decreased expression of all the TFs may definitely affect heart development and cause developmental defects in the heart. It has been documented that Agtr2, Tnnt3, Edn1, and Osr1 play crucial roles in the determination of left ventricle size, cardiac septation and function (70–72). Their decreased expression as observed in the present study is in accordance with the systolic/diastolic dysfunction and structural remodeling observed in echocardiography (Figure 2). Further, our enrichment analysis showed down-regulation of a number of genes, viz. Pf4, Gdf6, Sall1, Apln, Kctd11, and Twist2 have functions associated with the differentiation of cardiomyocytes, endothelial cells and striated muscles (66, 69, 73–76). In addition to differentiation, the regulation of proliferation in cardiomyocytes is important for heart development and its normal functions. Studies showed that the rate of cardiomyocyte proliferation is high during early embryogenesis; however, within the first 2 weeks after birth, cardiomyocytes exit the cell cycle, and the rate of cardiomyocyte proliferation continuously decreases (77, 78). We observed alteration of gene expression related to cell proliferation such as Tgfbr1 (79), Agtr2 and Kctd11. These data further supported the histological finding of the neonatal hearts of diabetic females (Figure 5B). Thus, hyperglycemia during PGDM may adversely impact cardiogenesis as well as cell differentiation and proliferation. Propagation of the electrical impulse for coordinated contractions is performed by the cardiac conduction system in the heart. Transcription factors, for example, the pacemaker gene Bmp4 and the Pitx2 play an important role in the development of the sinoatrial node (SAN). Pitx2 also mediates the right-sided development of the SAN by inhibiting left-sided pacemaker specification (80, 81). In our study, we found a decrease in the expression of the above two genes that are associated with the regulation of the cardiac conduction system. The data corroborates our electrocardiography results that indicate the alteration of electrophysiology of the heart in the neonates from diabetic female rats (Figure 1). The observed cardiac plasticity in neonates from the diabetic mother might be due to transgenerational inheritance through an epigenetic mechanism which leads to alteration in gene expression pattern in offspring from diabetic parents (82). Various studies have shown that there is a link between hyperglycemia in T2DM and epigenetic modification such as DNA methylation and post-translational modifications (e.g., *N*-Glycosylation and acetylation) (83, 84).

Myocardial fibrosis is a common histopathologic finding in CHDs and has been associated with arrhythmias and decreased cardiac functions. Collagen deposition occurs in response to various stimuli such as hypertension, ischemia, diabetes, and aging. The gene Spp1 is involved in myocardial remodeling by facilitating the formation of insoluble collagen (85). Recent findings show that BMP10 has an anti-fibrotic role by regulating SMAD- and STAT3 signaling pathways (86). The decoy type I receptor, Bambi, antagonizes TGF-β signaling that causes uncontrolled extracellular matrix deposition and protects the heart from aberrant myocardial fibrosis (87). Decreased expression of these genes as observed in the present study is consistent with histological findings (Figure 5A). In the neonatal hearts of diabetic rats, alteration in expression of the genes Slit3, Pbx1, Cd4, Nr4a1, and Dach1 (Table 1) suggests that coronary artery growth, patterning of great arteries, and angiogenesis may also be affected in the neonatal heart (57, 62, 88).

We used the human TF database as a background in the TRRUST software so that novel or crucial regulators of the DEGs in the context of human cardiovascular complications can be identified. Furthermore, these identified TFs can be used for future research or can serve as possible drug targets in the treatment of CHDs. As shown in Figure 6, one of the most significant regulatory relationships of DEGs was found with HIF1A, which plays an important role in all diabetic complications associated with diabetic pregnancy. HIF-1α is indispensable for normal embryonic development, and its decreased levels during hyperglycemia were associated with increased susceptibility to diabetic embryopathy (89). The highest number of affected genes in the neonatal heart due to PGDM was found with SP1, which is an essential TF for normal embryogenesis (90). Changes in SP1 expression and its post-translational modification are associated with pathologic cardiac remodeling *in vivo* (91). TGF-β signaling plays a central role in cardiac fibrosis and is activated by TGFBR1, followed by its differential regulation by SMADs like SMAD2, SMAD3, and SMAD4 (92, 93). Regulatory interactions with the SMADs further support increased fibrosis in neonates from diabetic mothers (Figure 5). Thus, modulation of the affected TF genes/proteins will be promising to address the problem associated with PGDM-induced early neonatal heart development. For example, HDACs interact with and modulate various cardiac development-related TFs such as GATA, NFAT, and MEF2 (94). Manipulating the activity of TF identified in our study by using HDAC inhibitors and thus acetylation level might modulate TF-binding and respective gene expression programs.

The strength of our study is reflected by the fact that the phenotypic changes are significantly correlated with the modulation of multiple genes in the associated pathway and the study highlights molecular defects in offspring as a result of pre-existing diabetes in the mother. However, the limitation of our study is that we have validated only a few genes for their expression at RNA and protein levels in neonatal hearts. Also, we have not validated the gene regulatory network analysis for their physical interaction by ChIP-Seq, for example. Moreover, our study is focused on analyzing cardiac defects due to PGDM only at the early stage of neonates. Studying the same correlation in neonates at later stages of life will provide more understanding of the progression of cardiac abnormalities.

## Conclusion

**FIGURE 7:**
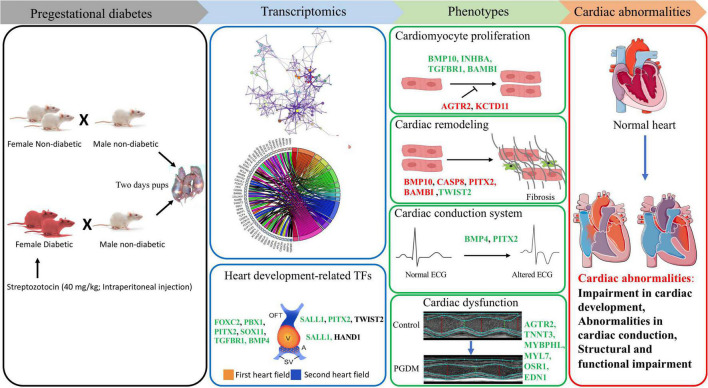
Correlation of transcriptomic perturbation during PGDM with the phenotypic changes. Transcriptomics analyses, including differential gene expression and pathway analysis of the neonatal hearts from STZ-induced diabetic rats, demonstrated that PGDM leads to the downregulation of genes related to heart development, structure and function. The alteration in gene expression also affects cardiomyocyte proliferation, cardiac remodeling, electrophysiology and echocardiographic parameters, leading to cardiac abnormalities. Upregulated genes are colored black, the genes having a positive effect on the indicated pathway are colored green, and the genes having a negative impact on the indicated pathway are colored red.

Our findings from the gene expression and gene ontology studies unequivocally correlate with the phenotypic studies (Figure 7). The findings of our study reveal the cardiac pathophysiology of neonates born from pregestational diabetic mothers and help to identify the therapeutic targets for PGDM-associated cardiac complications. In this study, we have demonstrated the altered electrophysiology and impaired systolic function in the hearts of neonates born from STZ-induced diabetic female rats. Our RNA sequencing analyses suggest that alteration in the expression of the genes involved in the development, differentiation and electrical conduction of the hearts in the offspring of mothers experiencing pre-existing diabetes and thus reflect an underlying structural and functional plasticity of the heart.

## Data availability statement

The data presented in this study are deposited in the Gene Expression Omnibus (https://www.ncbi.nlm.nih.gov/geo/query/acc.cgi?acc=GSE196242) repository, accession number: GSE196242.

## Ethics statement

The animal study was reviewed and approved by Institutional Animal Ethical Committee (IAEC) of Translational Health Science and Technology Institute (THSTI), Faridabad-India or National Institute of Pharmaceutical Education and Research (NIPER), Guwahati.

## Author contributions

MA: conceptualization, investigation, writing – original draft, review and editing, formal analysis, software, and visualization. SU: investigation, validation, writing – original draft, review and editing, and visualization. VT, BV, and SM: investigation. RA: resources. SA: investigation and validation. SB: supervision, conceptualization, methodology, writing – review and editing, funding acquisition, resources, and project administration. All authors read and approved the final manuscript.

## Footnotes

1
https://rgd.mcw.edu/

2
www.metascape.org

3
http://david.ncifcrf.gov

4
https://www.grnpedia.org/trrust/

## Supplementary material

Supplementary Figure 1 **(A)** Average base quality (C1 and D1 samples). The position in the read is plotted on the *x*-axis, and the *Q*-score is plotted on the *y*-axis. The red line is the median value *Q*-score. The dark blue line is the mean value *Q*-score. The boxplot represents the interquartile range, while the whiskers represent the 10 and 90% points. A *Q*-score above 30 (>99.9% correct) is considered high-quality data. **(B)** Summary of mapping and unmapping reads by samples. The graph showing percentage of the reads mapped to the Rattus norvegicus reference genome for each sample of control and PGDM groups. **(C)** Overview of pipeline used in the RNA-seq analysis. **(D)** A volcano plot showing the relationship between fold change and *p*-values. A volcano plot showing the relationship between log2 fold change and *p*-values is plotted for differentially expressed transcripts. Regions of interest in the plot: (1) Those points are found toward the top of the plot (high statistical significance), (2) Points at extreme left or right (strongly down and up-regulated, respectively).

Supplementary Figure 2 **(A)** H&E images of heart tissues from neonatal rats from control and diabetic animals (40x) showing frequent mitosis (arrows) and the measurement of cardiomyocyte size in longitudinally sectioned cells. Data are shown as Mean ± SEM (*n* = 3), ^∗∗^*p* < 0.01. **(B)** Representative gel images and densitometry showing expression levels of Sox11, Mmp9, and Rpl32 genes by PCR. Data are shown as Mean ± SEM (*n* = 5), ^∗∗∗^*p* < 0.001, ns, non-significant. **(C)** Western blot images and their densitometry showing expression levels of Pitx2, Pbx1, Foxo3a, and Sox11 genes. The stain-free gel was used as a loading control. Data are shown as Mean ± SEM (*n* = 3), ^∗^*p* < 0.05, ^∗∗^*p* < 0.01. **(D)** Confirmation of diabetes induction in female rats. Intraperitoneal administration of streptozotocin in female SD rats and subsequent level of blood glucose both in control and diabetic groups. Data are shown as Mean ± SEM (*n* = 8), ^∗∗∗∗^*p* < 0.0001.

Supplementary Table 1 Read numbers and mapping results for the eight RNA-sequencing libraries.

Supplementary Table 2 List of parameters of echocardiographic measurements (M-mode and PWD) in control and diabetic neonatal rats.

Supplementary Table 3 Full name, log2(fold change) and p values of important genes related to cardiac structure and development were found in the study.

## Abbreviations

BP: biological process
CHD: congenital heart defects
DEG: differentially expressed genes
DGE: differential gene expression
ECG: electrocardiogram
FC: fold change
FDR: false discovery rate
GDM: gestational diabetes
GO: gene ontology
GTex: genotype-tissue expression
LVAW: left ventricular anterior wall thickness
LVAWd: LVAW at end-diastole
LVAWs: LVAW at end-systole
LVIDs: left ventricular internal diameter at end-systole
LVOT VTI: left ventricular outflow tract velocity time integral
LVPW: left ventricular posterior wall thickness
LVPWd: LVPW at end-diastole
LVPWs: LVPW at end-systole
MF: molecular function
PGDM: pregestational diabetes
PWD: pulsed-wave doppler
RNA-seq: RNA sequencing
RPKM: read per kilobase of exon per million mapped reads
SAN: sinoatrial node
STZ: streptozotocin
TF: transcription factor
TRRUST: transcriptional regulatory relationships unraveled by sentence-based text-mining.
