# Supplementary material for: Pregestational diabetes alters cardiac structure and function of neonatal rats through developmental plasticity
Source: Front Cardiovasc Med. 2022 Sep 13;9:919293. doi: 10.3389/fcvm.2022.919293 (PMC9514058; doi:10.3389/fcvm.2022.919293)
Supplement: Supplementary Figure 1 — (A) Average base quality (C1 and D1 samples). The position in the read is plotted on the x-axis, and the Q-score is plotted on the y-axis. The red line is the median value Q-score. The dark blue line is the mean value Q-score. The boxplot represents the interquartile range, while the whiskers represent the 10 and 90% points. A Q-score above 30 (>99.9% correct) is considered high-quality data. (B) Summary of mapping and unmapping reads by samples. The graph showing percentage of the reads mapped to the Rattus norvegicus reference genome for each sample of control and PGDM groups. (C) Overview of pipeline used in the RNA-seq analysis. (D) A volcano plot showing the relationship between fold change and p-values. A volcano plot showing the relationship between log2 fold change and p-values is plotted for differentially expressed transcripts. Regions of interest in the plot: (1) Those points are found toward the top of the plot (high statistical significance), (2) Points at extreme left or right (strongly down and up-regulated, respectively). [file Presentation_1.PPTX]

## Slide 1
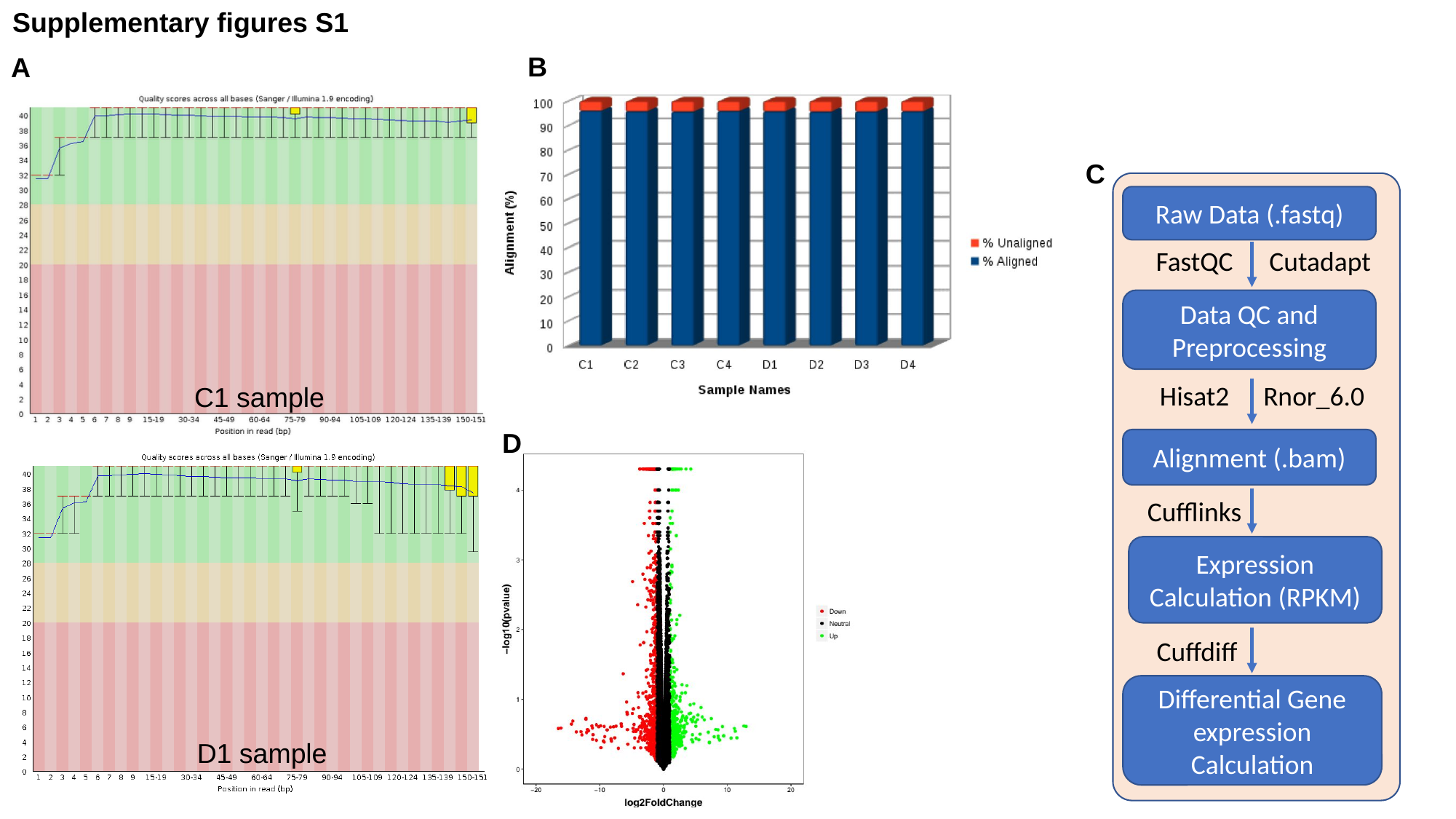

Supplementary figures S1
B
A
C1 sample
D1 sample
C
Raw Data (.fastq)
FastQC
Cutadapt
Data QC and Preprocessing
Rnor_6.0
Hisat2
Alignment (.bam)
Cufflinks
Expression
Calculation (RPKM)
Cuffdiff
Differential Gene expression
Calculation
D

## Slide 2
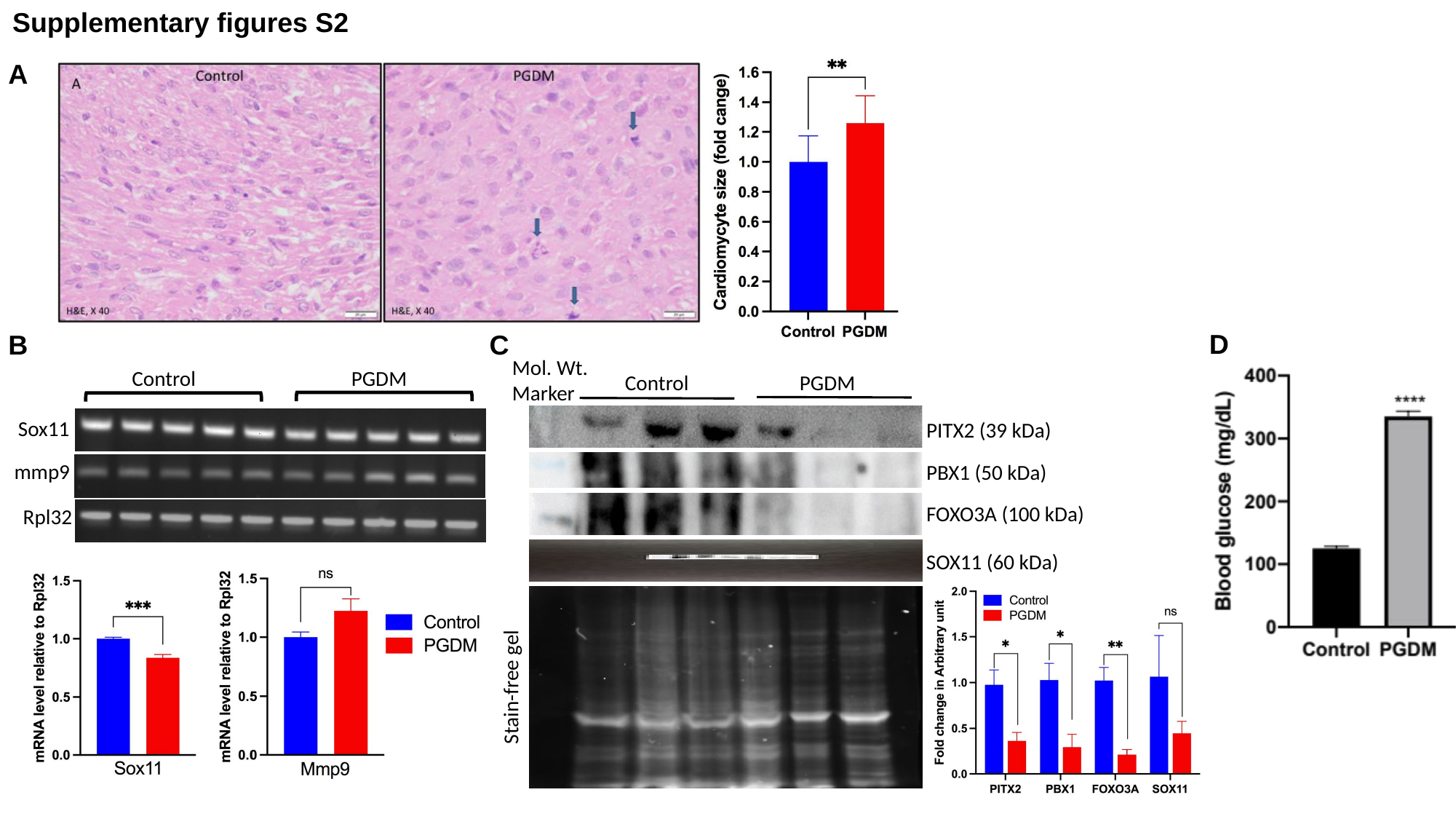

Supplementary figures S2
A
D
C
B
Control
PGDM
Sox11
mmp9
Rpl32
Mol. Wt.
Marker
Control
PGDM
PITX2 (39 kDa)
PBX1 (50 kDa)
FOXO3A (100 kDa)
SOX11 (60 kDa)
Stain-free gel
