# Supplementary material for: Pregestational diabetes alters cardiac structure and function of neonatal rats through developmental plasticity
Source: Front Cardiovasc Med. 2022 Sep 13;9:919293. doi: 10.3389/fcvm.2022.919293 (PMC9514058; doi:10.3389/fcvm.2022.919293)
Supplement: Supplementary Table 1 — Read numbers and mapping results for the eight RNA-sequencing libraries. [file Table_1.pdf]

Table S1. **Read numbers and mapping results for the eight RNA-sequencing libraries**

Sample ID	*Raw Reads (in million)*	*Processed Reads (in million*	*% of High Quality Data*	*% Aligned*	*% Unaligned*	*Transcript Expressed*	*Transcripts with >1 FPKM*
C1	27.996	26.064	93.101	96.1	3.9	22130	13822
C2	30.750	28.573	92.923	95.93	4.07	22799	14294
C3	25.287	23.539	93.085	95.72	4.28	21769	13537
C4	30.980	28.561	92.191	95.98	4.02	22598	14282
D1	32.910	30.668	93.186	95.93	4.07	22434	13790
D2	29.013	27.007	93.087	95.74	4.26	22078	13583
D3	26.558	24.574	92.530	95.82	4.18	22093	13740
D4	29.142	27.043	92.797	95.91	4.09	22296	13872
