# Supplementary material for: Pregestational diabetes alters cardiac structure and function of neonatal rats through developmental plasticity
Source: Front Cardiovasc Med. 2022 Sep 13;9:919293. doi: 10.3389/fcvm.2022.919293 (PMC9514058; doi:10.3389/fcvm.2022.919293)
Supplement: Supplementary Table 2 — List of parameters of echocardiographic measurements (M-mode and PWD) in control and diabetic neonatal rats. [file Table_2.pdf]

Table S2. List of parameters of echocardiographic measurements (M-mode and PWD) in control and diabetic neonatal rats.

	Control (n=6)	***Diabetic (n=6)***
LVIDd (mm)	2.325±0.24	1.828± 0.124**
LVIDs (mm)	0.969±0.142	1.223±0.223*
LVPWd (mm)	0.494±0.099	0.535±0.117
LVPWs (mm)	0.508±0.048	0.790±0.051****
LVAWs (mm)	1.070±0.129	1.072±0.072
LVAWd (mm)	0.448±0.074	0.655±0.034****
Volume (systolic) (µL)	2.222±0.644	3.275±1.5
Volume (diastolic) (µL)	19.04±4.92	11.627±0.951**
Stroke Volume (µL)	17.025±4.598	11.248±0.615*
Ejection Fraction (%)	89.285±3.830	78.177±7.876*
Cardiac Output (mL/min)	3.740±0.982	2.245±0.4**
LV Mass Corrected (mg)	18.835±4.022	18.627±3.311
Fractional Shortening (%)	58.188±5.749	45.295±7.072**
Heart rate (BPM)	220.33±22.82	262.06±11.44**
Mean velocity (mm/s)	490.02±141.74	330.70±46.87*
VTI (mm)	51.485±12.216	25.863±2.358***

***Note*:** Left ventricular internal diameter at systole (LVIDs) and at diastole (LVIDd), left ventricular posterior wall thickness at end systole (LVPWs) and at diastole (LVPWd), left ventricular anterior wall thickness at systole (LVAWs) and at diastole (LVAWd), Beats per minute (BPM), Velocity time integral (VTI). Data are presented as mean ± SD. n = 6/group. *$p < 0.05$ *vs* Control, **$p < 0.01$ *vs* Control, ***$p < 0.001$ *vs* Control, ****$p < 0.0001$ *vs* Control.
