# Supplementary material for: Pregestational diabetes alters cardiac structure and function of neonatal rats through developmental plasticity
Source: Front Cardiovasc Med. 2022 Sep 13;9:919293. doi: 10.3389/fcvm.2022.919293 (PMC9514058; doi:10.3389/fcvm.2022.919293)
Supplement: Supplementary Table 3 — Full name, log2(fold change) and p values of important genes related to cardiac structure and development were found in the study. [file Table_3.pdf]

Table S3. Full name, log2(fold change) and p-values of important genes related to cardiac structure and development found in the study.

Gene symbol	*Gene name*	*log2(fold change)*	*p-value*	*FDR (5%)*
Spp1	Secreted phosphoprotein 1	2.54976	0.00625	0.01594636
Atf3	Activating transcription factor 3	1.51833	0.00005	0.00050591
Nr4a1	Nuclear receptor subfamily 4, group A, member 1	1.42133	0.0003	0.00201445
Hand1	Heart and neural crest derivatives expressed 1	1.3215	0.0012	0.00564564
Dach1	Dachshund family transcription factor 1	1.31155	0.0001	0.00090627
Nr4a3	Nuclear receptor subfamily 4, group A, member 3	1.08745	0.00415	0.012116
Tfcp2l1	Transcription factor CP2-like 1	1.06604	0.00025	0.00181927
Slit3	Slit guidance ligand 3	-1.04019	0.00005	0.00050591
Nfatc4	Nuclear factor of activated T-cells 4	-1.07392	0.00175	0.00732364
Sall1	Spalt-like transcription factor 1	-1.12425	0.00005	0.00050591
Edn1	Endothelin 1	-1.17042	0.0009	0.00468155
Bcl6	B-cell CLL/lymphoma 6	-1.17599	0.00115	0.00551582
Inhba	Inhibin beta A subunit	-1.1853	0.00015	0.00126627
Agtr2	Angiotensin II receptor, type 2	-1.1904	0.0051	0.01405618
Foxc2	Forkhead box C2	-1.20735	0.0002	0.00154691
Hck	HCK proto-oncogene, Src family tyrosine kinase	-1.21375	0.01215	0.02377673
Cebpa	CCAAT/enhancer-binding protein alpha	-1.22259	0.0074	0.01743509
Bambi	BMP and activin membrane-bound inhibitor	-1.23573	0.00005	0.00050591
Twist2	Twist family bHLH transcription factor 2	-1.30535	0.01155	0.02308864
Pbx1	PBX homeobox 1	-1.32917	0.0372	0.04305818
Nppa	Natriuretic peptide A	-1.3836	0.00005	0.00050591
Casp8	Caspase 8	-1.50091	0.008	0.01835118
Pitx2	Paired-like homeodomain 2	-1.57657	0.00095	0.00478982

Bmp4	Bone morphogenetic protein 4	-1.58184	0.0005	0.002908
Kctd11	Potassium channel tetramerization domain containing 11	-1.59893	0.00005	0.00050591
Nr2f1	Nuclear receptor subfamily 2, group F, member 1	-1.65216	0.00005	0.00050591
Osr1	Odd-skipped related transcription factor 1	-1.65686	0.00005	0.00050591
Sox11	SRY box 11	-1.71031	0.00005	0.00050591
Gdf6	Growth differentiation factor 6	-1.97412	0.00005	0.00050591
Cldn1	Claudin 1	-2.01745	0.00005	0.00050591
Apln	Apelin	-2.07343	0.00005	0.00050591
Cx3cr1	C-X3-C motif chemokine receptor 1	-2.44761	0.00375	0.01142218
Bmp10	Bone morphogenetic protein 10	-3.682	0.00005	0.00050591
Tgfbr1	Transforming growth factor, beta receptor 1	-6.3363	0.04305	0.04665927
Tnnt3	Troponin T, fast skeletal muscle	-1.10794	0.0483	0.04917455
Cd4	T-cell surface glycoprotein CD4	-1.24101	0.00105	0.00518773
